# Staged revascularization and multi-modal mechanical circulatory supports in a patient with severe cardiogenic shock due to acute-on-chronic coronary syndrome

**DOI:** 10.1093/jscr/rjad631

**Published:** 2023-11-20

**Authors:** Miri Horimoto, Masahiro Tsutsui, Nobuhiro Mochizuki, Yuki Setogawa, Fumitaka Suzuki, Masahiko Narita, Aina Hirofuzi, Shingo Kunioka, Tomonori Shirasaka, Natsuya Ishikawa, Hiroyuki Kamiya

**Affiliations:** Department of Cardiac Surgery, Asahikawa Medical University, Asahikawa 078-8510, Hokkaido, Japan; Department of Cardiac Surgery, Asahikawa Medical University, Asahikawa 078-8510, Hokkaido, Japan; Department of Cardiac Surgery, Asahikawa Medical University, Asahikawa 078-8510, Hokkaido, Japan; Department of Cardiac Surgery, Asahikawa Medical University, Asahikawa 078-8510, Hokkaido, Japan; Department of Cardiac Surgery, Asahikawa Medical University, Asahikawa 078-8510, Hokkaido, Japan; Department of Cardiac Surgery, Asahikawa Medical University, Asahikawa 078-8510, Hokkaido, Japan; Department of Cardiac Surgery, Asahikawa Medical University, Asahikawa 078-8510, Hokkaido, Japan; Department of Cardiac Surgery, Asahikawa Medical University, Asahikawa 078-8510, Hokkaido, Japan; Department of Cardiac Surgery, Asahikawa Medical University, Asahikawa 078-8510, Hokkaido, Japan; Department of Cardiac Surgery, Asahikawa Medical University, Asahikawa 078-8510, Hokkaido, Japan; Department of Cardiac Surgery, Asahikawa Medical University, Asahikawa 078-8510, Hokkaido, Japan

**Keywords:** staging procedure, Impella 5.5, low cardiac shock

## Abstract

Acute coronary syndrome with cardiogenic shock is a life-threatening condition, but with planned staged treatment combined with coronary revascularization and mechanical circulatory supports its management is increasingly possible. Here, we present our successful life-saving case. A 76-year-old male patient was diagnosed with ST-elevation myocardial infarction with cardiogenic shock due to severe stenosis of the left main coronary artery based on the severe triple vessel disease. We initially introduced Impella CP and performed a percutaneous coronary intervention without stenting on the patient. We maintained hemodynamics with Impella CP and performed coronary artery bypass grafting after a week. Intraoperatively, Impella CP was left to function as a left ventricular vent. The patient required upgrading to Impella 5.5 plus veno-arterial extracorporeal membrane oxygenation postoperatively, but his condition gradually improved, all mechanical circulatory supports could be weaned off, and he eventually survived.

## Introduction

Acute coronary syndrome (ACS) is a life-threatening condition, and its treatment is extremely difficult especially in patients suffering from cardiogenic shock. However, in recent years, disease management, even in the most severely injured patients, has become possible through the concerted efforts of the heart team concept and through the combination of a step-by-step treatment plan and appropriate adjuvant circulatory management. Here, we describe a case of severe cardiogenic shock due to ACS that was successfully treated with staged coronary revascularization and several mechanical circulatory supports.

## Case report

**Figure 1:**
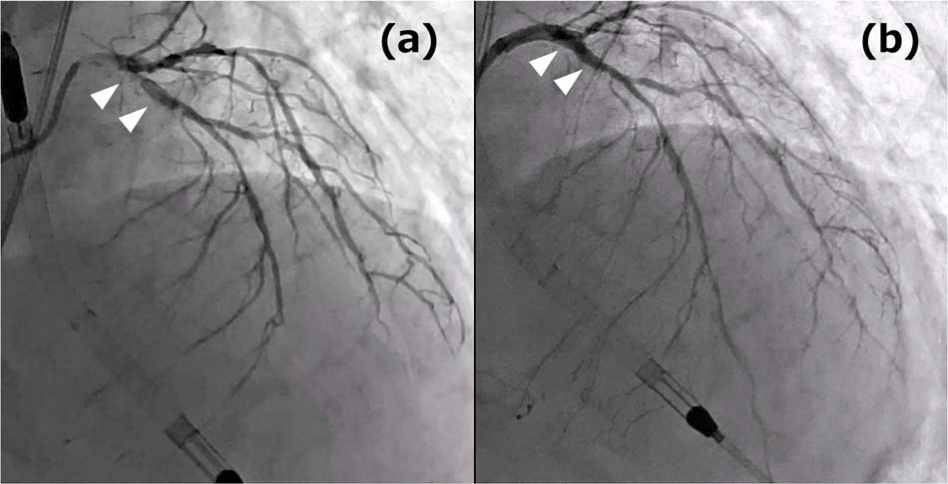
**(a)** Coronary angiography (CAG) before PCI: the white arrows indicate culprit lesions; **(b)** CAG post PCI: the white arrows indicate the treatment area.

**Figure 2:**
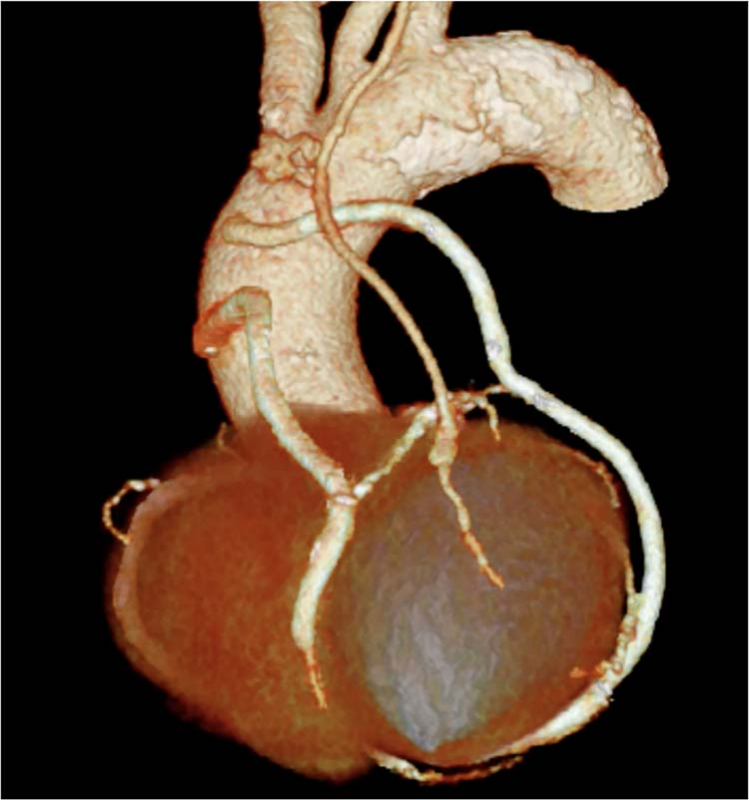
Postoperative 3D CT image showing the bypass graft design.

A 76-year-old male patient visited the emergency room with complaints of chest pain at rest. Upon hospital arrival, the patient was in cardiogenic shock (blood pressure: 68/48 mmHg, heart rate: 96 bpm). His blood samples showed elevated troponin I (5345.6 pg/ml) and a 12-lead electrocardiogram (ECG) revealed an ST-elevation in lead aVR. Therefore, the patient was diagnosed with ST-elevation myocardial infarction (STEMI), and emergency coronary angiography revealed severe stenosis of the left main coronary artery (LMCA). At that point, Impella CP (Abiomed, Danvers, MA, United States) was introduced from the left femoral artery. Subsequently, severe stenotic lesions were identified in the left anterior descending artery (LAD), the left circumflex artery, and the right coronary artery, and the patient was diagnosed with severe triple-vessel disease. Percutaneous coronary intervention (PCI) was performed only on the LMCA to LAD (Fig. 1), which appeared to be the culprit lesion and the 12-lead ECG revealed ST changes improvement. No other lesions were touched, and initial treatment ended at that point. However, other peripheral lesions remained, and transthoracic echocardiography revealed a remarkably reduced ejection fraction in this condition to 25%, thus revascularization of the remaining lesion was also considered essential. We maintained hemodynamics by continued circulatory support with Impella CP, and after improvement of general condition, we performed a standby elective coronary artery bypass graft (CABG) a week after the onset. CABG was performed with cardiopulmonary bypass without cardioplegic arrest (on-pump beating) and the graft design includes left internal thoracic artery-diagonal branch, great saphenous vein (SVG)–second diagonal branch–LAD, SVG-first obtuse marginal branch (OM)–second OM–atrioventricular node branch (Fig. 2). Impella CP was left to function as a left ventricular vent intraoperatively. We anastomosed the LAD by the SVG because it was a diffuse lesion with only a small area at the apex where anastomosis was possible, and we considered the perfusion area to be localized. The circulation of the patient became unstable on the first postoperative day; therefore, veno-arterial extracorporeal membrane oxygenation (ECMO) was introduced and he was managed with the so-called Ecpella (ECMO+Impella). The Impella CP was upgraded to Impella 5.5 from the right axillary artery on postoperative Day 4 because long-term Impella management was anticipated. Thereafter, his cardial function had gradually improved and he was weaned from ECMO on postoperative Day 6 and weaned from Impella 5.5 on postoperative Day 18. The patient’s general condition continued to improve gradually, and he was transferred to the hospital for continued rehabilitation on postoperative Day 68. One year after the operation, he is now doing well.

## Discussion

According to the American College of Cardiology/American Heart Association/Society for Cardiovascular Angiography and Interventions guidelines, in patients with STEMI and cardiogenic shock, routine revascularization of the nonculprit artery is class III: Harm. Elective CABG after heart team consultation is recommended as class IIa in terms of reducing the risk of cardiac events if there is residual complex multivessel nonculprit artery disease after successful PCI [1]. The patient was treated exactly guideline-oriented with the help of several mechanical circulatory supports.

Barringhaus *et al*. [2] reported the efficacy of staged revascularization for STEMI. They reported that patients who underwent staged PCI of residual disease after primary PCI had lower hospital mortality. They also reported that patients who underwent CABG for residual disease after primary PCI were less likely to be readmitted to the hospital and less likely to undergo unscheduled reintervention.

Impella CP was upgraded to Impella 5.5 in this case. The main advantage of upgrading large Impella is for long-term Impella management. Impella CP is intended to be used for 8 days, while Impella 5.5 is intended for 30 days. Other reported benefits include the possibility of reducing the risk of hemolysis, such as the ability to maintain a sufficient volume of assistance even with a small number of rotations [3]. There is an additional advantage of Impella 5.5 over Impella CP; because Impella 5.5 is inserted through the axillary artery, while Impella CP is inserted normally from the femoral artery, enhanced rehabilitation program in patients with Impella 5.5 may be possible.

As intraoperative circulatory supports, several previous studies have reported the use of Impella in off-pump CABG [4, 5]. However, in this case, CABG was performed using cardiopulmonary bypass as on-pump beating fashion and the Impella CP was used as a left ventricular vent. Thus, comprehensive data on the intraoperative use of Impella remained unavailable, and their effectiveness needs to be further investigated.

## Conclusion

Staged revascularization with multi-modal mechanical circulatory supports may be an option in patients with severe cardiogenic shock due to acute-on-chronic coronary syndrome.
